# Betrayed by the nervous system: a comparison group study to investigate the ‘unsafe world’ model of selective mutism

**DOI:** 10.1007/s00702-021-02404-1

**Published:** 2021-08-14

**Authors:** Siebke Melfsen, Marcel Romanos, Thomas Jans, Susanne Walitza

**Affiliations:** 1 Department of Child and Adolescent Psychiatry and Psychotherapy, Psychiatric University Hospital Zurich, University Zurich, Neumunsterallee 3, P.O. Box 233, 8032 Zurich, Switzerland; 2 Center of Mental Health, Department of Child and Adolescent Psychiatry, Psychosomatics and Psychotherapy, University Hospital of Wurzburg, Würzburg, Germany

**Keywords:** Selective mutism, Aetiology, High sensory-processing sensitivity, Dissociation, Anxiety, Schoolchildren

## Abstract

**Abstract:**

The study presented in the following verifies some assumptions of the novel ‘unsafe world’ model of selective mutism (SM). According to this model, SM is a stress reaction to situations erroneously experienced via cognition without awareness as ‘unsafe’. It assumes a high sensitivity to unsafety, whereby the nervous system triggers dissociation or freeze mode at relatively low thresholds. We examine whether there is a correlation between SM, sensory-processing sensitivity and dissociation. We compared a sample of 28 children and adolescents with SM (mean age 12.66 years; 18 females) to 33 controls without SM (mean age 12.45 years; 21 females). Both groups were compared using a medical history sheet, the ‘Selective Mutism Questionnaire’ (SMQ), a ‘Checklist for Speaking Behaviour’ (CheckS), the ‘Highly Sensitive Person Scale’ (HSPS), the ‘Child Dissociative Checklist’ (CDC), the ‘Adolescent Dissociative Experience Scale’ (A-DES) and the ‘Social Phobia and Anxiety Inventory for Children’ (SPAIK). Appropriate parametric and non-parametric tests were conducted to examine differences between groups. The results indicate that sensory-processing sensitivity was significantly higher in the group of children and adolescents with SM [*X*^2^(1) = 7.224, *p* = 0.0007; *d* = 1.092]. Furthermore, dissociative symptoms were more common in children and adolescents with SM than in controls [*F*(1, 33) = 13.004, *p* = 0.001; *d* = 0.986]. The results indicate that sensory-processing sensitivity and dissociation are important factors of SM that may hold important implications for the treatment.

**Trial Registration:**

This study is registered with the ClinicalTrials.gov number NCT04233905.

## Background

Selective mutism (SM) is characterized by an absence of speech in selected situations in which children are expected to speak, although a physical disability to speak is not present (American Psychiatric Association 2013). However, in other situations they speak quite normally, e.g., to immediate family members or close friends with whom they feel comfortable. Usually they have most difficulty at school, nursery or kindergarten and in unfamiliar social situations. Lack of physical distance to other people has also been found to be an important trigger for SM behaviour (Schwenck et al. 2021). The disorder usually begins in transitional situations from parental home to kindergarten and elementary school (Muris and Ollendick 2021). According to epidemiological studies, SM is a relatively rare disorder with a prevalence rate of around 1% (Muris and Ollendick 2015).

Besides the predominant symptom of silence, there are additional symptoms in SM. These include a noticeable reduction in the use of facial expressions or gestures (e.g., Johnson and Wintgens 2015), as well as cramped-looking postures with limited movements. Some children with SM seem to freeze (e.g., Hill and Scull 1985). Gaze aversion may be observed (e.g., Dobslaff 2005, p. 28). Additionally, abnormal subjective experience of their own voice is reported (Black and Uhde 1992; Boon 1994) as well as reduced function of auditory reflexes (Arie et al. 2007; Bar-Haim et al. 2004; Muchnik et al. 2013).

Several studies have found evidence for an association between SM and clinically significant social anxiety as well as other anxiety disorders (Muris and Ollendick 2015; Vogel et al. 2019; Schwenck et al. 2019). Accordingly, in DSM-5 (American Psychiatric Association 2013) SM is classified as an anxiety disorder. Cohan et al. (2006) assume that a child experiencing high levels of anxiety is particularly sensitive to verbal interactions. Muris and Ollendick (2015) concluded in their review about the relationship between SM and anxiety that both disorders tend to overlap in terms of aetiology, symptomatology, and treatment approaches.

However, according to a meta-analysis by Driessen et al. (2020), the precise nature of the relationship between SM and anxiety is still unclear. On self-report measures, children with SM do not show higher levels of anxiety compared with socially anxious children (Yeganeh et al. 2003, 2006; Melfsen et al. 2006). In their meta-analysis, Driessen et al. (2020) could show that 80% of the children with SM were diagnosed with an additional anxiety disorder. Among this group, 19% were diagnosed with a specific phobia like a fear of flying which is not sufficient to explain SM. Moreover, the remaining 20% lacked an additional anxiety diagnosis. The authors emphasize that the presence of a comorbid anxiety disorder does not necessarily imply that SM originates from the same source. Consequently, they conclude that it is not clear whether anxiety plays a key role in SM.

Several further findings support the assumption that SM cannot fully be explained by anxiety, e.g.,

- Heilman et al. (2012) reported that children and adolescents with SM show a dampened autonomic reactivity during mobilization which is unique to individuals with SM. Young et al. (2012) confirm that contrary to socially anxious children, children with SM experience *less arousal* during social interaction tasks. They do not demonstrate heightened levels of arousal above and beyond those of children with social anxiety.
- Steinhausen and Juzi (1996) and Muris and Ollendick (2015) report that a background of *speech and language impairment* and delayed *motor development* is quite common in SM. There are no corresponding findings for social anxiety.
- The incidence of SM seems to be high among children of *immigrant* families (Elizur and Perednik 2003). Similar results are not reported for social anxiety.
- Children and adolescents with SM describe an *abnormal subjective experience of their own voice* (Black and Uhde 1992; Boon 1994; Arie et al. 2007; Bar-Haim et al. 2004; Muchnik et al. 2013). That has not been reported for social anxiety.
- In contrast to social anxiety, children with SM show reduced function of *auditory reflexes* and seem to ignore other people’s normal voices (Arie et al. 2007; Bar-Haim et al. 2004; Muchnik et al. 2013).
- SM may even affect *immediate* family members with whom communication previously had been possible (e.g., Steinhausen and Juzi 1996). This finding can hardly be explained by anxiety.
- The *contact behaviour* of children and adolescents with SM is described not only as cautious but as dismissive. They avoid contact and sometimes even react aggressively to contact offers (e.g., Ballnik 2009, p. 80). The children and adolescents with SM themselves often describe a passive state of absence with little action control and low body awareness. That is not the case in anxious children. A study of Nowakowski et al. (2011) investigated joint attention behaviours between parents and children with SM or other anxiety disorders. The results showed that children with SM—but not with other anxiety disorders—withdrew from interactions with their parents and were less responsive to them. This behaviour led to a break down in the parent–child communication.
- Communication partners of children and adolescents with SM often feel *provoked*, helpless and disappointed (Bahr 2004, pp. 65–66, p. 76; Hartmann 1997, p. 40; Katz-Bernstein 2005, p. 21). Responses to anxious children are described as caring and protective, to teach coping and problem-solving skills (Nowakowski et al. 2011).
- Besides the discussion of oppositional-like behaviour in SM (Vecchio and Kearney 2005; Carbone et al. 2010; Cunningham et al. 2004; Levin-Decanini et al. 2013), several parents describe *violent tantrums* and diverse *sibling conflicts* at home (Hartmann 1997) while anxious children tend to show inconspicuous behaviour at home.

In summary, the most common explanation for SM is an underlying anxiety disorder. However, there are several findings that cannot fully be explained by anxiety. Consequently, other factors that might play a role in the aetiology of SM should be included in research (Driessen et al. 2020).

**Fig. 1:**
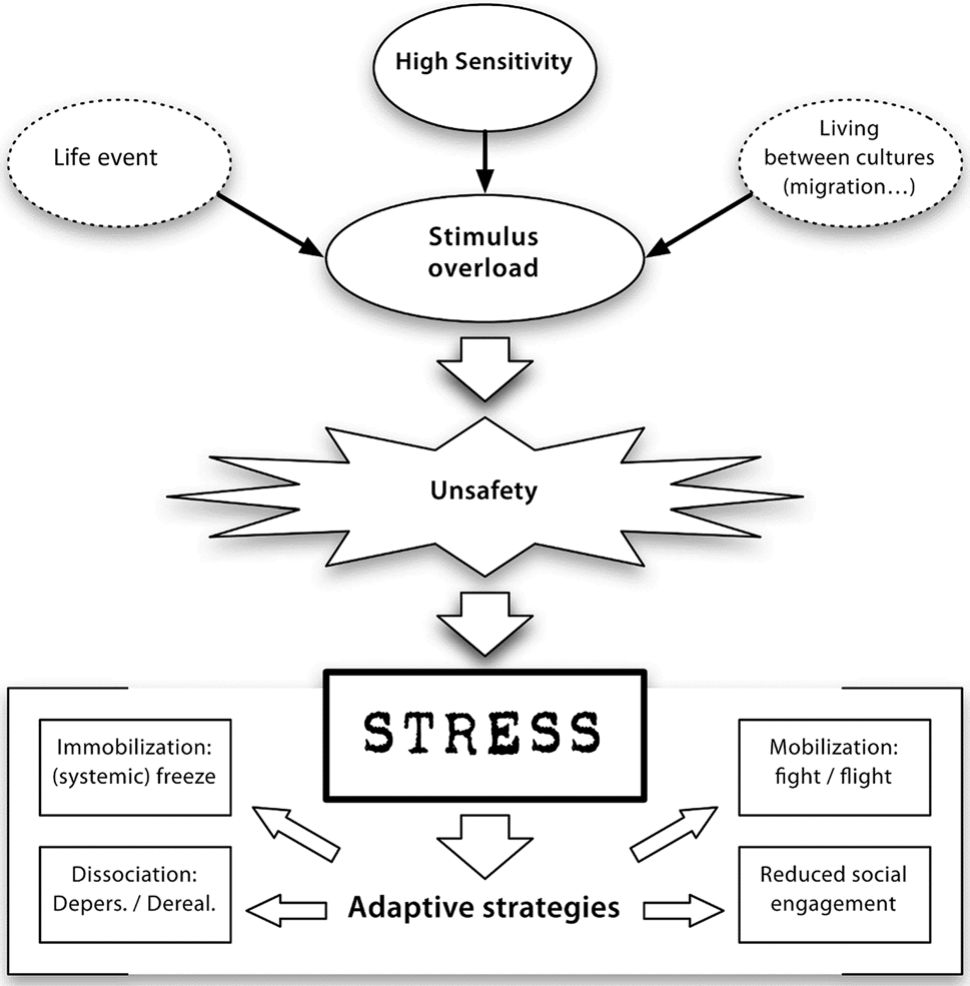
The unsafe world model

In our ‘unsafe world’ model (Fig. 1), we postulate that SM is an automatic stress reaction in situations erroneously classified via cognition without awareness as ‘unsafe’. If a situation is experienced as unsafe, it does not actually have to be unsafe to trigger an alarm and subsequently a stress reaction. There are several contributors that may be involved in the mediation, e.g., *high sensitivity*. The question whether a certain situation is considered safe or unsafe depends, among other factors, on the sensory-processing sensitivity of each individual. We assume that many individuals with SM have an extraordinary high sensitivity to external and internal sensory stimuli. As a consequence, the nervous system of children with SM readily reacts, e.g., to weak signs of unfamiliarity or lack of physical distance and classifies the situation as ‘unsafe’. The high sensory-processing sensitivity thus causes a stress reaction in situations that normally do not require a stress reaction.

In a stress reaction, the nervous system can switch to *dissociation*. Whereas pathological dissociation often involves experiences of trauma, normal transient dissociation is considered to be relatively common in childhood and adolescence and may also occur in the face of psychologically overwhelming circumstances like powerful emotional events (Putnam 1997). Consequently, dissociation can be regarded as a coping mechanism to tolerate stress. Repeated similar experiences further increase the habituation to the non-speaking behaviour. The experience of being unable to speak in certain situations can lead to anxiety as a secondary disorder.

Thus, the shutting down of social engagement of children with SM may be an adaptation to a situation that the nervous system has “erroneously” evaluated as unsafe. In this situation, a nonverbal state gets adopted. Thus, in some way, those who suffer from SM may be ‘betrayed by their own nervous system’.

In our study, we examine two assumptions of our model. First, whether children and adolescents with SM show a higher sensory-processing sensitivity; second, whether they show a reduced threshold for dissociative experience compared with a control group without SM.

## Methods

### Procedure

In our study, we compared a sample of children and adolescents with SM (MG) to controls without SM (CG). The study was approved by the local research ethics committees (94/18-me, BASEC-No. 2017-00679) at all participating sites. It was undertaken according to the Declaration of Helsinki and Good Clinical Practice Principles. Interested parents and children were given comprehensive information. Informed consent was obtained from all individual participants included in the study. The study was registered with the ClinicalTrials.gov NCT04233905.

The study comprised paper-and-pencil questionnaires to be filled out by the mother as well as by the child at home. A combination of in-person and postal mail administration was employed. On average, 1 h was needed for the children as well as for their mothers to answer all given questions. Parents mailed the questionnaires back to the investigators in a self-addressed, postage-paid envelope.

### Participants

The sample consisted of 28 participants with SM, living in Germany and Switzerland. They were recruited at the Department of Child and Adolescent Psychiatry and Psychotherapy of the University Hospital of Zurich, the Department of Child and Adolescent Psychiatry, Psychosomatics and Psychotherapy of the University of Wurzburg and the Mutism Special Outpatient Clinic of the University of Dortmund. Furthermore, they were recruited in collaboration with two non-profit advocacy groups that maintain an informational website (https://www.mutismus.de and https://stille-staerken.de), as well as through leaflets in psychotherapeutic outpatient practices. The children and adolescents were eligible for inclusion if they met DSM-5 diagnostic criteria for SM according to the information provided by the mothers in the medical history sheet (Melfsen 2014) in addition to a diagnosis previously made by a psychiatrist or psychologist and a diagnostic cut-off of the ‘Selective Mutism Questionnaire’ (SMQ). The children and adolescents with SM and their mothers also had sufficient command on the German language to complete the questionnaires. Exclusion criteria were a history of previously diagnosed communication disorders or autism spectrum disorder that could better account for the child’s symptoms (Table 1).

**Table 1:** Demographics

	Selective mutism (SM)	Control group
	(*n* = 28)	(*n* = 33)
	*M* (SD)	*M* (SD)
Age	12.66 (3.98)	12.45 (3.18)
Age of SM onset:	3.24 (1.26)	
Age of SM diagnosis:	7.70 (4.28)	
Duration of SM:	9.04 (4.44)	
Sex (female/male)	(18/10)	(21/12)
Mother tongue: German	25	32
Twin sibling	3	0
Physical or sexual abuse	5	0
Life events	11	8
Comorbid diagnoses		
Anxiety	4	0
Depression	4	0
ADS/ADHS	1	2
Read spelling disorder	1	0
Developmental specifics during infancy and toddler age		
Motor developmental delay	5	0
Speech developmental delay	3	0
Emotional problems	4	0
Sleeping problems	3	1

The participants were aged 7–18 years. The average age was 12.66 years (SD = 3.98) and 18 of the participants were female. The majority’s mother tongue was German (89.29%). This sample was compared to a control group without SM recruited through leaflets. Mothers were asked for psychiatric abnormalities which would have excluded children from participation in the study. All controls were offered a small compensation for participation. A total of 33 control participants aged 7–18 years took part in the study. The average age of the control group was 12.45 years (SD = 3.18). Twenty-one of the participants were female. The majority’s mother tongue was German (97%). Both groups were comparable in respect of age, gender, ethnicity and educational level (Table 1).

### Measures

The constructs of ‘selective mutism’, ‘dissociation’, ‘sensory-processing sensitivity’ and ‘social anxiety’ were measured using the following questionnaires. For all German translations forward/backward translation has been conducted:

*Selective Mutism* In a medical history sheet the mothers of the children with SM first made some demographic statements and gave information on the child`s course of SM (Melfsen 2014). Symptoms of SM, its severity, scope and functional impairment were assessed by the German version of the parent report ‘Selective Mutism Questionnaire’ (SMQ) (Bergman et al. 2008; Letamendi et al. 2008; Oerbeck et al. 2020). The SMQ is a 17-item parent-rating measure designed to assess the severity of SM. The test uses a 4-point scale to rate the frequency of the child’s speaking behaviour in school (6 items), home/family (6 items) and public settings outside school (5 items), from 0 (“never”) to 3 (“always”) for each, with a total score of 51. The German version of the measure has shown satisfactory internal consistency (α = 0.83–0.96; *N* = 179) with total scale reliability of α = 0.95. Furthermore, the SMQ total values of a group of 96 children and adolescents with SM and 80 children and adolescents without SM were compared. The SMQ total value differed significantly between the group with SM (*M* = 19.08; SD = 7.49) and the group without SM [*M* = 42.39; SD = 8.71; *t*(0.95, 169) = 18.88; *p* < 0.001; *d* = 2.90]. The factorial structure of three factors of the English version has been confirmed. The SMQ diagnostic cut-off is *M* ≤ 2 (Melfsen and Walitza in preparation).

Further information regarding symptom severity was gained using a checklist for speaking behaviour, parent report (CheckS) (Melfsen 2014). It is designed to assess the communicative burden of various socially interactive situations for children with SM on a five-point scale (from 0 = “never” to 4 = “always”) by 53 items. A distinction is made between different contexts, namely the persons to be spoken to, the type of communication, the length of the spoken answers, the conversational situations, the contents of the conversation, the places and surroundings, the expectations of those present and the unpredictability of contexts. The total score divided by item number ranges from 0 to 4.

*Sensory-processing sensitivity* The German version of the ‘Highly Sensitive Person Scale’ (HSPS) (Aaron (2002, 2012; pp. 17–18) was used to assess sensory-processing sensitivity. In high sensory-processing sensitivity, individuals perceive and process external and internal stimuli more intensely than the average population. The test is a parent-report questionnaire with 23 items to be answered on a seven-point Likert scale (from 1 “not at all” to 7 “absolutely”). The total score divided by item number ranges from 0 to 7. The German version provides approximately normal distributed data. The reliability value of Cronbach’s α = 0.87 (*N* = 179) is good. The factorial structure of the English version has been confirmed.

*Dissociation* The German translation of the ‘Child Dissociative Checklist’ (CDC; Putnam 1997) is a parent report of 20 items using a three-point Likert scale (0 = “not true”; 1 = “somewhat true”, 2 = “very true”). Parents are asked to report dissociative behavioural problems of their child within the past 12 months. The scores of all items are summed together and the total score ranges from 0 to 40. A cut-off score of 12 or more is considered to indicate clinical levels of dissociation. For the German version a reliability of α = 0.82. (*N* = 179) was determined.

The children and adolescents completed the ‘Adolescent Dissociative Experience Scale’ (A-DES; Armstrong et al. 1997; Carlson and Putnam 1993) to assess dissociative symptoms. Thirty items are to be assessed on an 11-point rating scale (from 0 = “never” to 10 = “always”) in terms of their frequency. The total score divided by item number ranges from 0 to 10, with a cut-off score for clinical levels of dissociation of 4 or more. For the German version total scores’ reliability of α = 0.82 (*N* = 179) was determined. Furthermore, a high correlation with the CDC (rho = 0.37, *p* < 0.01) has been shown.

*Social anxiety* The German version of the ‘Social Phobia and Anxiety Inventory for Children’ (SPAIK; Beidel et al. 1995; Melfsen et al. 2001) is a self-describing inventory used to assess social anxiety in children and adolescents. The questionnaire consists of 26 situations assessing somatic, cognitive and behavioural aspects of social anxiety. They measure characteristic aspects of social anxiety on a three-point Likert scale (from 0 = “never or seldom” to 3 = “most of the time or always “) with a total score of 52. Internal consistency of the normal sample is 0.92 (Cronbach’s alpha). Retest reliability after 2 weeks is *r*_tt_ = 0.85, after four weeks *r*_tt_ = 0.84. In the clinical sample the internal consistency is α = 0.95. Validity has been confirmed by factorial structure and criteria validity.

### Statistical analyses

The statistical programming package R (R Core Team 2020) was used for data analyses. The sample size was calculated for *t* test/variance analyses procedure with a significance level of *p* < 0.05 and power of 0.90. This procedure led to a required sample size of 20 children/adolescents per group. Assuming a drop-out rate of 15% (3), we calculated a sample size of 23 per group. Prior to conducting statistical analyses, variables were screened for accuracy of data entry, normal distribution, and missing values. The required minimum completion for partially filled questionnaires was set to 90%.

In three cases, appropriate non-parametric tests were conducted to examine any differences between groups due to the data being not normally distributed. In two cases, a *t* test was conducted.

## Results

*Speaking behaviour* Questionnaire data from the SMQ demonstrated that children and adolescents with SM (*n* = 28) scored low, which is indicating high intensity of SM symptoms (*M* = 19.64, SD = 7.62). In the control group (*n* = 33), the score was significantly higher with a mean of *M* = 42.76 (SD = 11.28). The median test showed a significant group difference [*X*^2^(1) = 29.697, *p* = 0.0001]. The effect intensity was *d* = 2.191, indicating a strong effect (Table 2).

**Table 2:** Results

	Selective mutism	Control group	Effect
	(*n* = 28)	(*n* = 33)	
	*M* (SD)	*M* (SD)	
SMQ	19.64 (7.62)	42.76 (11.28)	*d* = 2.19
Check-S	2.05 (0.54)	3.07 (0.59)	*d* = 1.79
HSPS	4.85 (0.98)	3.76 (1.01)	*d* = 1.09
CDC	5.77 (6.03)	1.42 (2.28)	*d* = 0.98
A-DES	2.18 (1.57)	1.17 (1.06)	*d* = 0.77
SPAIK	30.72 (7.48)	10.34 (7.98)	*d* = 2.63

*SMQ* Selective Mutism Questionnaire, *CheckS* Checklist for Speaking Behaviour, *HSPS* Highly Sensitive Person Scale, *CDC* Child Dissociative Checklist, *A-DES* Adolescent Dissociative Experience Scale, *SPAIK* Social Phobia and Anxiety Inventory for Children

*Check-S* Examination of the sample means indicated that the group with SM (*M* = 2.05, SD = 0.54) scored significantly less than the control group [*M* = 3.07, SD = 0.59; *t*(0.95, 58) = 6.99, *p* < 0.001]. The effect intensity *d* = 1.785 was strong.

*Sensory-processing sensitivity* Mothers rated children and adolescents with SM as significantly more sensitive than the control group [*X*^2^(1) = 7.224, *p* = 0.0007]. Whereas the mean of the group with SM was *M* = 4.85 (SD = 0.98), the mean of the control group was 3.76 (SD = 1.01). The effect intensity *d* = 1.092 was strong.

*Dissociative symptoms* According to data obtained using the CDC, the children and adolescents of the group with SM were rated by their mothers as having significantly more symptoms of dissociation (*M* = 5.77, SD = 6.03) than the control group [*M* = 1.42, SD = 2.28; *F*(1, 52) = 6.62; *p* < 0.01]. The Welch test showed significant group differences [*F*(1, 33) = 13.004, *p* = 0.001], with a strong effect intensity of *d* = 0.986.

The reported dissociation symptoms by the children and adolescents themselves on the A-DES showed similar results. Comparisons revealed that the group with SM (*M* = 2.18, SD = 1.57) scored significantly higher than the control group (*M* = 1.17, SD = 1.06). The Welch test showed significant group differences [*F*(1, 40) = 7.467, *p* = 0.009] with a medium effect intensity of *d* = 0.765.

*Social phobia* Significant group differences were found when comparing the SPAIK means. The group with SM showed significantly higher means (*M* = 30.72, SD = 7.48) than the control group (*M* = 10.34, SD = 7.98; *t*(0.95, 52) = − 9.844; *p* < 0.001) with a strong effect intensity of *d* = 2.627.

*Medical history sheet* Differences between the group with SM and the control group were observed in the number of twin siblings. There were three twins in the group of SM and no twin siblings in the control group. Eleven mothers (39.3%) of children with SM reported significant life events (such as family conflicts, physical health problems, job loss, home or country change). Five mothers reported abuse of their children. Eight mothers of the control group (24.2%) reported significant life events; none reported abuse of their children.

## Discussion

The present study was undertaken as a first step to evaluate a novel model of SM: the ‘unsafe world’ model. In this model, we postulate that SM is a stress reaction to a situation erroneously experienced via cognition without awareness as ‘unsafe’. As an unconscious process, it enables humans to engage in social behaviours by distinguishing safe from unsafe contexts (Porges 2011).

The assumption that SM is caused by a stress reaction is supported by neurobiological research. Porges (2003; 2011) has done extensive research on the ‘Polyvagal Theory’. The Polyvagal Theory focuses on the structure of two efferent branches of the vagus nerve for emotion regulation. It links physiological states to different classes of stress strategies like fight, flight and freeze behaviour as well as to spontaneous social engagement behaviours. The vagal system works in opposition to the sympathetic-adrenal system. The Polyvagal Theory describes how the autonomic nervous sub-systems are linked to three areas of behaviour:

- social communication (e.g., facial expression, vocalization, listening),
- mobilization (e.g., fight–flight behaviours), and
- immobilization (e.g., shut down or dissociation).

Only if we feel safe enough, it is possible to engage in social connectedness, including making eye contact, listening and talking to people. Inability to speak, poor gaze, low facial expressivity, stiff body postures, limited motor behaviours, changed awareness of the sound of the human voice, are all symptoms of SM that can be explained by a stress reaction. According to the Polyvagal Theory, children and adolescents with SM have difficulties in re-establishing safe, calm states that would promote normal social communication (Heilmann et al. 2012). Instead, their bodies are in constant stress mode. High sensitivity to external stimuli like noise and lights as well as internal stimuli like ingested food may all be emergent properties of this physiological state of stress (Porges 2010).

Various factors can influence the process of distinguishing safe from unsafe contexts (Porges, 2011). In our study, for example, five mothers of the SM group stated that their child had experienced physical or sexual abuse; none of the mothers in the control group reported any abuse. A study by MacGregor et al. (1994) showed corresponding results. Experiencing abuse and maltreatment during childhood has life-long health consequences (e.g., Boeck et al. 2016). It causes stress and may lead to the perception of unsafety. However, recent studies did not show a clear link between trauma and SM (Muris and Ollendick 2015). This may stem from the assumption that abuse is only one of several risk factors for SM.

Following the vulnerability model for SM (Steinhausen and Juzi 1996), the development of SM is supported by speech, motor and emotional abnormalities in early childhood. Some of these unspecific risk factors also show up in our study (Table 1). Families of a child with abnormal development face more stressors such as behavioural difficulties, health concerns, and thus increased contact with health and mental health services as well as educational placement difficulties (Blacher et al. 2005). That is why parenting stress has been found to be higher amongst parents of children with developmental disorders than in those with healthy children (Gerstein et al. 2009; Woodman 2014). Parental stress increases the perceived unsafety of their children. Moreover, there are some hints connecting autonomic dysregulation with speech impairment (e.g., Jones et al. 2014), motor impairment (e.g., Zamuner et al. 2011) or alexithymia (e.g., Neumann et al. 2004). Therefore, the atypical regulation found in the autonomic system of children with SM (Heilman et al. 2012) could explain the higher incidence of speech and motor impairment.

It is also remarkable that there were three pairs of twins in the SM group of our study. This may indicate that pregnancy and childhood as a twin may be an unspecific risk factor increasing stress. An alternative explanation for the high prevalence for twins in the SM group could be that twins typically have a very close link to each other that may lead to a strong contrast between the ‘safe’ inner wold and ‘unsafe’ outer world.

SM is also more prevalent in migrants (Elizut and Perednik 2003), where stress from the migration situation, from experiencing two cultures and overcoming strangeness/unfamiliarity is prevalent. Additionally, a migration background itself may divide the environment more easily into ‘safe’ and ‘unsafe’ situations.

According to the ‘unsafe world’ model, an important influencing factor is the sensitivity for external and internal sensory stimuli. The nervous system of individuals with SM appraises the environment as being ‘unsafe’ even when it is ‘safe’. As a consequence, their physiological state does not support social engagement behaviours. According to Polyvagal Theory (Porges 2011), sensory-processing sensitivity is not genetically determined, but a function of the current physiological state of stress. The results from our study support our hypothesis of high sensory-processing sensitivity. The results show that sensory-processing sensitivity is significantly higher in the group of children and adolescents with SM. This result is in line with our assumption that the autonomous nervous system of children and adolescents with SM may already react to comparably low signs of ‘unsafety’. As a consequence, dissociation or the freeze mode gets activated. In this mode, a nonverbal state takes over and social engagement is shut down which is compellingly in line with Polyvagal Theory (Porges 2011). SM may be an adaptation to a situation that the nervous system has evaluated erroneously as ‘unsafe’. Feeling erroneously ‘unsafe’ in a ‘safe’ situation, children and adolescents with SM are in a way “betrayed” by their own nervous system.

Repeated stress experiences like being unable to speak in selective situations increases dissociation and leads to habituation of non-speech. Our study shows that dissociative symptoms are more common in children with SM than in controls. Dissociation is not only pathological but a ubiquitous reaction that serves adaptive and defensive purposes. Repeated similar experiences may continuously reduce the threshold for dissociative experience. In general, children are more dissociative than adults with a peak at about 9–11 years of age and a decline during adolescence. Children of that age typically have a greater vulnerability to develop dissociative disorders (Putnam and Peterson 1994).

The results of our study show significantly higher social anxiety in the group of SM than in controls. Following the ‘unsafe world’ model, anxiety may also be a secondary response to SM. This is in line with a new meta-analysis of Driessen et al. (2020). They doubt the current conceptualization of SM as an anxiety disorder. According to their data, anxiety is not always present in SM. The authors conclude that it is yet uncertain how SM should be classified and recommend to broaden the scope of factors which might be relevant for aetiology.

The nature of the relationship between SM and behavioural inhibition has been investigated by Gensthaler et al. (2016). Behavioural inhibition is defined as a tendency to withdraw, to inhibit play and vocalization and to seek a parent in unfamiliar situations (Kagan et al. 1990). Behavioural inhibition can be observed in 10–15% of the population (Kagan 1994). This significantly exceeds the prevalence of SM. Behavioural inhibition is a well-known unspecific risk factor for the development of other disorders, especially for anxiety disorders. Behavioural inhibition thus may be an unspecific risk factor for developing SM, as it is for other disorders, too. But behavioural inhibition is not sufficient as a sole precondition for developing SM.

There are many heterogeneous symptoms observed in SM. Several of these symptoms are consistent with a stress reaction rather than with anxiety:

- In a study by Young et al. (2012), children with SM did not show increased physiological arousal during social interaction tasks. In contrast to socially anxious children, they showed less physiological arousal. Low arousal can well be explained by Polyvagal Theory that stipulates that SM is associated with a dampened response of the vagal brake with reduced reactivity in heart rate and respiration and inability to activate the structures involved in speech (Heilmann et al. 2012). As a consequence, low physiological arousal during social interaction tasks is in line with a stress reaction.
- Children and adolescents with SM report abnormal subjective experience of their own voice and show reduced function of auditory reflexes (Arie et al. 2007; Bar-Haim et al. 2004; Thomas et al. 1985). This observation can well be explained by the assumption that the children and adolescents with SM are in stress mode. During this mode, the muscles in the inner ear can contract in the event of sudden stress and amplify low-pitched hearing functions (Porges 2003). This may explain why children with SM seem to ignore other people’s normal voices and report changed perceptions of their own voice.
- Children and adolescents with SM show dismissive contact behaviour and seem to be indifferent (Ballnik 2009; Nowakowski 2011). If stress increases, the nervous system can switch to dissociation where individuals appear to be indifferent and disconnected. Being mute, turning the head away, dropping the eyes—these are signals of ‘not being present’ and ‘being disconnected’. Children and adolescents with SM often describe that they feel like a ‘little ghost’ or a ‘phantom’, which implies ‘not being connected’.
- Sometimes children and adolescents with SM do not even speak to certain family members (Steinhausen and Juzi 1996). This may be caused by high sensitivity towards the slightest sign of ‘unsafety’ in conversation including, e.g., the family member’s prosody. The following stress reaction may include dissociation. Because dissociation is increasingly amplified by repeated similar experiences, the stress reaction once established may maintain the behaviour of SM occurring in communication with familiar persons with whom the children with SM have previously spoken. In contrast, anxious children generally do not develop anxiety towards family members.
- The interaction with children and adolescents with SM often leads the communication partners to feel provoked (Bahr 2004; Hartmann 1997; Katz-Bernstein 2005). While anxious children may react non-verbally to be supported, children with SM show less reaction, which causes irritability. This may best be explained by a dissociation reaction. Dissociation is often misinterpreted as defiant refusal. But this reaction is outside of free will decision.
- Several parents describe violent tantrums and diverse sibling conflicts of their children and adolescents with SM at home (Hartmann 1997). The stress-sensitive nervous system alerts children with SM to trivialities and can trigger increased fight readiness, which may lead to frequent sibling disputes and oppositional behaviour.

Thus, the model may explain several findings of children and adolescents with SM, which could hardly be explained by anxiety. Furthermore, the model addresses the finding that not all children with SM report anxiety.

The present study has some limitations. First of all, the present results do not show a causal relationship but a correlation between SM, sensory-processing sensitivity and dissociation. The possibly underlying psychophysiological model by Porges (2011) was not investigated directly either. In addition, due to the low prevalence of SM, it is difficult to recruit a large sample of children with the disorder. Therefore, a replication of our data with a larger sample would be important to confirm the significant results of the present study. Furthermore, the sample of this study is a pre-teen group with mean age of 12.66 years while SM presents much earlier. Therefore, their symptoms may be more severe and persistent. Another limitation concerns the diagnostic and screening measures applied in the study: both groups were compared using parent-report and self-report questionnaires, but no other informants with different roles and prospects according to the multi-informative assessment were considered.

Despite several shortcomings, the present study is the first attempt to investigate a novel theoretical model and to enhance our understanding of a challenging disorder. More attention needs to be paid to the importance of high sensory-processing sensitivity, dissociation and stress reaction in the development and maintenance of SM.

Additional research is needed to compare children and adolescents with SM and anxious children in respect to physiological measures like heart rate measures, high sensory-processing sensitivity and dissociative symptoms.

## Conclusions

Our study presents the ‘unsafe world’ model of SM that tries to broaden the scope of factors which may be relevant for aetiology. According to this model, SM is a stress reaction to situations experienced via cognition without awareness as ‘unsafe’. High sensory-processing sensitivity to unsafety lowers the threshold when the nervous system triggers an activation of dissociation or the freeze mode. In our study, we examined whether there is a correlation between SM, high sensory-processing sensitivity and dissociation. The results indicate that sensory-processing sensitivity is significantly higher in the group of children and adolescents with SM. Furthermore, dissociative symptoms are more common in children with SM than in controls.

We believe that our results are relevant for a new comprehension of SM. The ‘unsafe world’ model integrates existing data and eliminates inconsistencies. Should the results of our study be confirmed, they could have far-reaching consequences. Once the caregivers understand that the behaviour of children and adolescents with SM is caused by high stress levels, their relationship may significantly improve. Instead of being aggrieved by the disturbing behaviour, more understanding and compassion may be possible.

If confirmed, our findings may also have important clinical implications for therapy. The majority of cognitive–behavioural interventions for SM were designed based on anxiety treatment. Focusing only on the reduction of anxiety may not be sufficient to treat SM. Following our findings, an important focus of therapy should be to improve self-regulation to bring stable balance into the nervous system. A broader intervention addressing high sensory-processing sensitivity, dissociative behaviours and parent training may be beneficial. It seems very important to ensure that the children and adolescents with SM feel safe. Therefore, providing cues to calm the autonomic nervous system appears to be very important.

## Data Availability

The dataset analysed during the current study is not publicly available.

## Abbreviations

A-DES: Adolescent Dissociative Experience Scale
CheckS: Checklist for Speaking Behaviour, Parent Report
HSPS: Highly Sensitive Person Scale
SMQ: Selective Mutism Questionnaire
SPAIK: Social Phobia and Anxiety Inventory for Children
